# Transcriptional Regulation and Transport of Terpenoid Indole Alkaloid in *Catharanthus roseus*: Exploration of New Research Directions

**DOI:** 10.3390/ijms18010053

**Published:** 2016-12-28

**Authors:** Jiaqi Liu, Junjun Cai, Rui Wang, Shihai Yang

**Affiliations:** 1 College of Chinese Herbal Medicine, Jilin Agricultural University, Changchun 130047, China; liujiaqi0817@163.com; 2 Crop Research Institute, Sichuan Academy of Agricultural Sciences, Chengdu 610066, China; 3 West China Hospital, Sichuan University, Chengdu 610066, China; cai.junjun@foxmail.com

**Keywords:** terpenoid indole alkaloids, biosynthesis, transcription factor, transporter, regulatory network, compartmentation

## Abstract

As one of the model medicinal plants for exploration of biochemical pathways and molecular biological questions on complex metabolic pathways, *Catharanthus roseus* synthesizes more than 100 terpenoid indole alkaloids (TIAs) used for clinical treatment of various diseases and for new drug discovery. Given that extensive studies have revealed the major metabolic pathways and the spatial-temporal biosynthesis of TIA in *C. roseus* plant, little is known about subcellular and inter-cellular trafficking or long-distance transport of TIA end products or intermediates, as well as their regulation. While these transport processes are indispensable for multi-organelle, -tissue and -cell biosynthesis, storage and their functions, great efforts have been made to explore these dynamic cellular processes. Progress has been made in past decades on transcriptional regulation of TIA biosynthesis by transcription factors as either activators or repressors; recent studies also revealed several transporters involved in subcellular and inter-cellular TIA trafficking. However, many details and the regulatory network for controlling the tissue-or cell-specific biosynthesis, transport and storage of serpentine and ajmalicine in root, catharanthine in leaf and root, vindoline specifically in leaf and vinblastine and vincristine only in green leaf and their biosynthetic intermediates remain to be determined. This review is to summarize the progress made in biosynthesis, transcriptional regulation and transport of TIAs. Based on analysis of organelle, tissue and cell-type specific biosynthesis and progresses in transport and trafficking of similar natural products, the transporters that might be involved in transport of TIAs and their synthetic intermediates are discussed; according to transcriptome analysis and bioinformatic approaches, the transcription factors that might be involved in TIA biosynthesis are analyzed. Further discussion is made on a broad context of transcriptional and transport regulation in order to guide our future research.

## 1. Introduction

Plant secondary metabolites are often produced either in certain tissues or cells under stress conditions or induced by various developmental, hormonal and environmental cues. The biosynthesis of secondary metabolites is often tightly regulated at transcriptional levels. Even in a plant cell where secondary metabolites are synthesized, the multiple enzymes and their reactions are often compartmentalized into various subcellular organelles, such as chloroplast, endoplasmic reticulum (ER), vacuoles, as well as apoplastic spaces. It is also often observed that the synthetic site of secondary metabolites is usually different from the site where these chemicals function, as defensive compounds against insects, bacteria or fungal pathogens. Thus, efficient transport of precursors, metabolic intermediates and the end products for biosynthesis, storage or function, are of critical in whole plant secondary metabolism and their biological significance. Many studies support the idea that transporters, either across membranes or intra-cellular trafficking, or inter-cellular long distance transport, provide another layer of regulation for metabolic flux [1,2]. Furthermore, these transmembrane transporters, vesicle trafficking components, as well as protein carriers are also regulated at transcriptional levels [3]. Therefore, transcriptional and transport regulation of secondary metabolite biosynthesis are critical research topics.

*Catharanthus roseus* (Madagascar periwinkle) is a perennial herb belonging to the family Apocynaceae. It produces over 100 different terpenoid indole alkaloids (TIAs), some of which exhibit strong pharmacological activities and are essentially used in clinical treatment of various diseases [4]. Vinblastine and vincristine, which have been used clinically to treat cancers since 1950s, are the most valuable dimeric TIAs in *C. roseus* [5]. These two dimeric TIAs are produced in trace amount in *C. roseus* by coupling vindoline and catharanthine, both of which have also been reported with anti-bacterial activities, anti-diabetic properties and diuretic actions [6]. Other TIAs from *C. roseus* such as ajmalicine and serpentine are used in anti-hypertensive and anti-neuro-inflammatory agents [6]. Due to extremely low yield of the highly valuable vinblastine and vincristine, substantial efforts in past decades have put on large-scale cell culture, bioreactor processing biotechnology and metabolic engineering to improve their production in order to meet the increasing demands from the market. However, the success is very limited. It has been realized that understanding of the TIA biosynthesis, transport and their regulation may empower our ability to apply new and robust molecular and genetic tools in metabolic engineering of the production of these valuable metabolites [7,8,9,10]. Several reviews were published about the organ-, tissue- and cell-specific as well as transcriptional regulation of TIA biosynthesis [8,11]. However, the breakthroughs made recently on TIA biosynthesis regulation and particularly, the intra- and inter-cellular TIA transport, from molecular biology, genomic and transcriptomics points of view have provided significant insights into these important dynamic cellular processes [12]. This review is to summarize these most recent progresses made on TIA biosynthesis, transcriptional regulation and their transport and to employ transcriptome analysis for further looking for TIA transcriptional regulators that are potentially involved in the TIA biosynthesis.

## 2. The Biosynthetic Pathway of the TIAs in *Catharanthus roseus*

**Figure 1:**
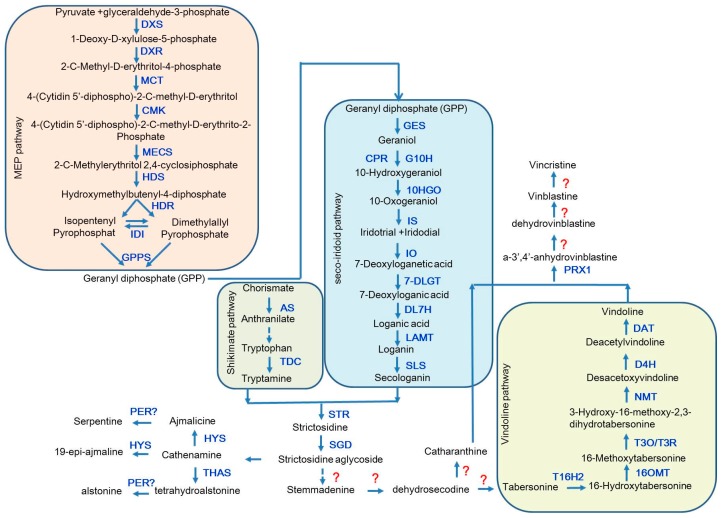
Schematic biosynthetic pathways for terpenoid indole alkaloids (TIAs) in *Catharanthus roseus*. The updated TIA biosynthetic pathways are presented by incorporating the most recently published results. Question marks in red indicate unknown process or enzymes for the reactions. Abbreviations are: DXS, 1-deoxy-d-xylulose 5-phosphate synthase; DXR, 1-deoxy-d-xylulose 5-phosphate reductoisomerase; MCT, MEP cytidyltransferase; CMK, 4-(cytidine 5′-diphospho)-2-*C*-methyl-d-erythritol kinase; MECS, 2-*C*-methylerythritol 2,4-cyclodiphosphate synthase; HDS, hydroxymethylbutenyl 4-diphosphate synthase; HDR, hydroxymethylbutenyl 4-diphosphate reductase; IDI, isopentenyl diphosphate isomerases; IPP isomerase; GPPS, geranyl diphosphate synthase; GES, geraniol synthase; CPR, cytochrome P450 reductase; G10H, geraniol 10-hydroxylase; 10HGO, 10-hydroxygeraniol dehydrogenase; IS, iridoid synthase; IO, iridoid oxidase; 7-DLGT, 7-deoxyloganetic acid glucosyltransferase; DL7H, 7-deoxyloganic acid hydroxylase; LAMT, loganic acid methyltransferase; SLS, secologanin synthase; AS, anthranilate synthase; TDC, tryptophan decarboxylase; STR, strictosidine synthase; SGD, strictosidine β-d-glucosidase; T16H2, tabersonine 16-hydroxylase 2; 16OMT, 16-hydroxytabersonine-16-*O*-methyltransferase; T3O, tabersonine 3-oxygenase; T3R, tabersonine 3-reductase; NMT, *N*-methyltransferase; D4H, desacetoxyvindoline 4-hydroxylase; DAT, acetyl CoA: deacetylvindoline 4-*O*-acetyltransferase; CR, cathenamine reductases; THAS, tetrahydroalstonine synthase; HYS, heteroyohimbine synthase; PRX1, a-3′,4′-anhydrovinblastine synthase; PER, putative peroxidase.

In recent decades, *C. roseus* has been extensively studied for elucidating the complex biosynthetic pathways of TIAs and now it has become an ideal medicinal plant for in-depth investigating the complex molecular mechanisms for TIA biosynthesis and transport as well as their transcriptional regulation. All the TIAs in *C. roseus* are derived from the central precursor strictosidine, which is a condensed product of the tryptophan pathway-derived tryptamine and the seco-iridoid pathway-derived secologanin by strictosidine synthase (STR) (Figure 1). Studies on the biosynthetic pathways of tryptamine and secologain have been carried out for years. Two enzymes have been revealed for their critical roles in tryptamine biosynthesis, anthranilate synthase (AS) and tryptophan decarboxylase (TDC) [7,13], however, specific process remains to be determined. In seco-iridoid pathway, secologanin is finally generated through eight steps after the hydrolysis of geranyl diphosphate (GPP) to geraniol by geraniol synthase (GES) [14], in such pathway, all the enzymes involved have been identified and the reversible reaction from 10-oxogeranial to 7-deoxyloganetic acid has also been illuminated, while the recent study did not found the intermediate product iridotrial in this reaction [15,16,17]. Subsequently, the central precursor strictosidine is converted into strictosidine aglycoside by strictosidine β-d-glucosidase (SGD) [18], and strictosidine aglycoside can be used to synthesize various kinds of TIAs. Crystal structure studies detected two new cathenamine reductases (CRs), namely, heteroyohimbine synthase (HYS) and tetrahydroalstonine synthase (THAS). HYS reduces cathenamine into ajmalicine and 19-epi-ajmalicine, while THAS is predominantly responsible for the conversion of tetrahydroalstonine from strictosidine aglycon [19]. Alstonine and serpentine are the oxidation products of tetrahydroalstonine and ajmaline respectively. Although the specialized oxidation enzyme has not been isolated, the oxidation conversion from ajmaline to serpentine present has been observed in plant vacuoles and is mainly accumulated in roots [20]. Vindoline is an important biosynthetic precursor of vincristine and vinblastine. Since the cytochrome P450 enzyme CYP71D1V2 tabersonine3-oxygenase (T3O) and an alcohol dehydrogenase (ADHL), tabersonine3-reductase (T3R) have been identified, the biosynthesis process from tabersonine to vindoline is well understood. However, the enzymes converting the strictosidine-aglycone into tabersonine remain to be identified. On the other hand, the whole biosynthesis of another precursor catharanthine remains to be revealed, although its putative intermediate, stemmadenine, has been identified for a long time [21]. After the biosynthesis of vindoline and catharanthine, they are coupled into dimeric alkaloids, a-3′,4′-anhydrovinblastine (AVLB), with the peroxidase a-3′,4′-anhydrovinblastine synthase (PRX1) [22]. The valuable TIAs in *C. roseus*, vinblastine and vincristine, were eventually derived from AVLB through multiple steps, which remain to be undetermined.

## 3. Transcriptional Regulation of TIA Biosynthesis

Plenty of evidence indicates that the synthesis of TIAs is strictly regulated by transcription factors that target on the key structural genes. Elicitors such as yeast, jasmonate (JA) and related oxylipins, hormones such as auxins as well as environmental cues can stimulate TIA biosynthesis in *C. roseus* [23,24]. It has been revealed that these stimuli-promoted TIA biosyntheses happen via transcriptional regulation of TIA synthetic genes, such as *STR*, *TDC*, *G10H*. The mechanisms for regulating the biosynthesis of different TIA end products are complicated and diversified; many transcription factors have been characterized for their regulatory functions and some of them have been successfully applied for TIA production [24].

There are many examples that applied different transcription factors to improve the content of plant secondary metabolites. Overexpression of *TSAR1* or *TSAR2* in basic helix-loop-helix (bHLH) gene family in *Medicago truncatula* hairy roots caused the up-regulation of triterpene saponin biosynthetic genes and increased accumulation of triterpene saponins [24]. A novel APETALA2/Ethylene Response Factors (AP2/ERF) family transcription factor PsAP2 from *Papaver somniferum* was overexpressed in tobacco, which showed improved resistance to both abiotic and biotic stresses [25]. After expressing seven MYB transcription factors (Dof1.1, IQD1-1, MYB28, MYB29, MYB34, MYB51 and MYB122) involved in aliphatic and indolic glucosinolate (GSL) biosynthesis in Chinese cabbage, the aliphatic and indolic GSL contents are changed [26]. Overexpression of AaERF1 and AaERF2 from *Artemisia annua* could increase the accumulation of artemisinin and artemisinic acid in transgenic *A. annua* plants [27]. Thus, it is an effective way to apply effective transcription factors for plant secondary metabolic engineering.

**Figure 2:**
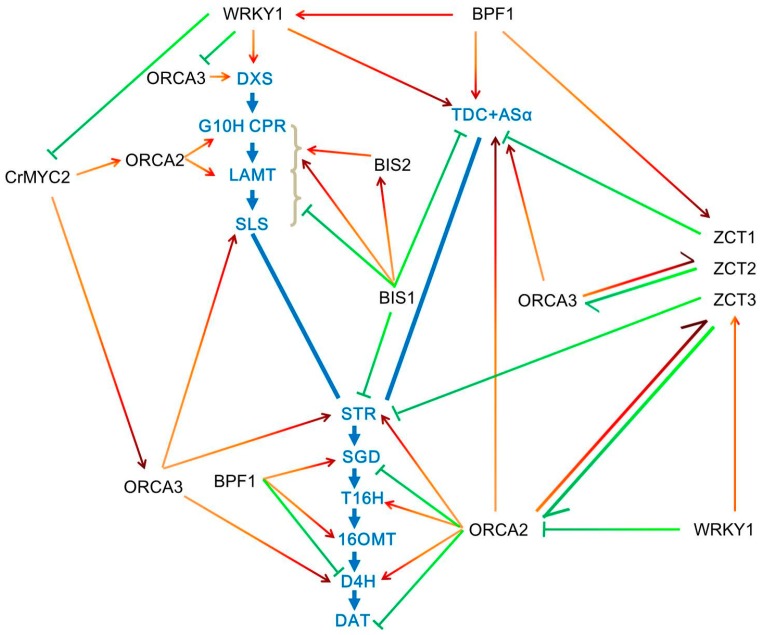
Regulation of TIA biosynthetic pathways by transcription factors in *Catharanthus roseus*. Various structural genes (marked in blue) in TIA biosynthetic pathway are regulated by different transcription factors (TFs). ORCAs are believed to be key regulators that directly bind to the promoters of structural genes involved in TIA biosynthesis. Several TFs such as WRKYs not only control the expression of structural genes but also regulate the expression of other TFs including MYC2, ORCAs, ZCTs, etc. Some TFs such as MYC2 could not directly regulate the expression of structural genes in biosynthetic pathways. Therefore, these TFs form a transcriptional regulatory network for TIA biosynthetic pathways. Among them, some TFs, such as BIS2, ZCT2 and WRKY2 (marked in green), are negative regulators, whereas most TFs (marked in red) are positively regulate TIA biosynthesis. DXS, 1-deoxy-d-xylulose 5-phosphate synthase; G10H, geraniol 10-hydroxylase; CPR, cytochrome P450 reductase; LAMT, loganic acid methyltransferase; SLS, secologanin synthase; STR, strictosidine synthase; SGD, strictosidine β-d-glucosidase; CR, cathenamine reductases; T16H2, tabersonine 16-hydroxylase 2; 16OMT, 16-hydroxytabersonine-16-*O*-methyltransferase; T3O, tabersonine 3-oxygenase; T3R, tabersonine 3-reductase; NMT, *N*-methyltransferase; D4H, desacetoxyvindoline 4-hydroxylase; DAT, acetyl CoA: deacetylvindoline 4-*O*-acetyltransferase.

Two AP2/ERF family transcription factors ORCA2 and ORCA3 were characterized as the critical regulators for TIA biosynthesis. They could specifically bind to the JERE (jasmonate and elicitor-responsive element) in *STR* promoter and can be up-regulated by JA [28]. In *C. roseus* hairy roots, the up-regulated expression of *ORCA2* significantly changed the transcripts of many structural genes in TIA biosynthesis, such as *AS*, *TDC*, *G10H*, *LAMT*, *STR*, *T16H*, *PRX1*, *D4H*, *SGD* and *DAT*; moreover, the induced *ORCA2* also caused the changes of the expressions of several TF encoding genes, such as *ORCA3*, *ZCT1*, *ZCT2*, *ZCT3* and *CrMYC2* (Figure 2). Accordingly, the accumulation of catharanthine, ajmalicine, serpentine and tabersonine were also changed dramatically after *ORCA2* induction [29]. Another study found that vindoline in the *ORCA2* transgenic hairy roots could be significantly increased compared to the control lines [30]. Therefore, ORCA2 plays an important role in the regulation of TIA metabolism. Another AP2/ERF factor ORCA3 was isolated by T-DNA activation tagging approach, which could also regulate the expressions of many TIA synthetic genes [31]. In *C. roseus* cell suspension cultures, overexpressing *ORCA3* caused the up-regulation of *TDC*, *STR*, *SLS*, *CPR*, *D4H*, *AS* and *DXS*, variable expression of *SGD*, as well as unaffected expression of *G10H* and *DAT*. Therefore, overexpression of *ORCA3* significantly induced tryptamine in shikimate pathway, while it did not cause secologanin in seco-iridoid pathway. Also, feeding loganin to *ORCA3*-overexpressed lines significantly increase TIA production [31]. Partly different from the results of *C. roseus* cell suspension cultures, overexpression of *ORCA3* and, along with JA elicitation in hairy root, induced the expression levels of *AS*, *DXS*, *SLS* and *STR*, decreased the expression of *SGD*, but not affected *TDC*, *G10H*, *CPR*, *GBFs* and *ORCA2*. The differences may result from different regulatory mechanisms between two types of tissue culture systems [32]. Therefore, the overexpression of *ORCA3* alone in two types of tissue cultures could not cover all the pathway genes, such as *G10H*, *SGD* and *DAT*. Another study shows that co-overexpression of *ORCA3* and *SGD* in *C. roseus* hairy roots resulted in a significant increase of many TIAs [33]. Co-overexpression of *G10H* and *ORCA3* in *C. roseus* plant and hairy roots both increased the accumulation of TIAs, indicating that co-overexpression of regulator and critical synthetic gene is an efficient way to increase metabolite production [34]. Besides JA, other ORCA3 inducers are found recently. Feeding artemisinic acid to *C. roseus* meristematic cells resulted in the increase of the transcript level of *ORCA3*, which indicated that artemisinic acid may be another way to induce ORCA3 [35]. Another study shows that inoculation of fungal endophytes in *C. roseus* could up-regulate the expression of *ORCA3* and enhance the accumulation of vindoline [36].

The fact that *ORCAs* can be induced by not only JA but also other elicitor implies the expressions of two *ORCA*s are regulated by more transcription factors, such as CrMYC2. As a positive regulator for the expressions of *ORCAs*, CrMYC2 was initially isolated through a yeast one-hybrid screening system using a tetramer of G-box from the *STR* promoter as bait, while it cannot control the active of the *STR* promoter [37]. CrMYC2 can regulate the expression *ORCA3* gene via binding to a specific sequence in the jasmonate-responsive element (JRE) from *ORCA3* promoter [38]. Overexpression or knockdown of *CrMYC2* significantly changed the expression of *ORCA*s and also had a strong effect on the accumulation of catharanthine and tabersonine, but showed no influence on *STR* and *TDC* expression, which indicated that CrMYC2 is critical for the expression of MeJA-responsive *ORCAs* and the accumulation of alkaloid but it could not regulate such structural genes as *STR* and *TDC* [39]. A bHLH transcription factor BIS1 from clade IVa isolated from *C. roseus* could regulate the expression of structural genes that ORCA3 cannot cover [40]. Overexpression of BIS1 in *C. roseus* cells and hairy roots caused a significant increase in the expression of the seco-iridoid pathway genes up stream of *LAMT* and the 2-*C*-methyl-d-erythritol-4-phosphate (MEP) pathway genes. Meanwhile, loganic acid, secologanin and strictosidine were highly accumulated in the BIS1-overexpressing cells. However, the accumulation of TIAs in transgenic hairy roots was not increased, which may be resulted from the down-regulation of ORCA3 target genes *LAMT*, *SLS*, *TDC*, and *SGD*. Decreased expression of the ORCA3 target genes was not caused by decreased expression of *ORCA2* or *ORCA3*, nor of any other known *C. roseus* TF encoding gene previously linked with regulation of the MIA pathway, and thus involves other, yet unknown, regulatory mechanisms. Another bHLH transcription factor BIS2 was identified recently, which is the homolog of BIS1 that can form homo- or heterodimers with BIS1. Same with BIS1, overexpressing *BIS2* in *C. roseus* suspension cells could also up-regulate MEP as well as seco-iridoid pathway genes, knockdown of *BIS2* completely abolished the JA-induced up-regulation of the seco-iridoid pathway genes and the accumulation of TIAs. The expression of *BIS2* could be regulated by BISs, while the BISs-binding sites of *BIS2* promoter remain to be determined. BIS1 and BIS2 could both regulate the structural genes that ORCA2 and ORCA3 cannot affect, such as *G10H* [41]. Therefore, BISs together with ORCAs may control the whole upstream pathway of TIA biosynthesis and support sufficient synthetic precursor of TIAs.

Two AP2/ERF proteins form a cluster with ORCA3, ORCA4 and ORCA5 were cloned, which are homologous to ORCA3. The expression of *ORCA4* and *ORCA5* can be also induced by JA like *ORCA3*. However, unlike *ORCA3*, *ORCA4* and *ORCA5* cannot be directly regulated by CrMYC2, they may be regulated by other transcription factors. Moreover, overexpression of *ORCA4* in *C. roseus* hairy roots significantly increased the transcripts levels of genes in both tryptophan pathway and seco-iridoid pathway, and also increased several TIAs, especially tabersonine. Therefore, ORCA4 is functionally overlapping but divergent with ORCA3 [42].

CrWRKY1, belonging to the group III WRKY superfamily, was identified in *C. roseus*, which can be induced by several phytohormones and preferentially expresses in roots [43]. Studies found CrWRKY1 regulates *TDC* by binding W box element in *TDC* promoter. Overexpression of *CrWRKY1* in *C. roseus* hairy roots increased several key pathway genes, such as *AS*, *DXS*, *SLS*, *SGD*, especially *TDC*; as well as TF encoding genes, such as *ZCTs*, while it represses the transcriptional activators *ORCA2*, *ORCA3*, and *CrMYC2*. Moreover, the accumulation of serpentine was significantly increased, while catharanthine was decreased. Therefore, CrWRKY1 is an ideal candidate to regulate serpentine branch to produce more ajmalicine and serpentine.

CrBPF1 is a kind of MYB transcription factor that binds to BA region in *STR* promoter. Overexpressing *CrBPF1* in *C. roseus* hairy roots changed the expression of many pathway genes. As for regulatory genes, overexpressing *CrBPF1* increased the transcripts of *ORCA3*, *CrMYC1*, *CrMYC2*, *BIS1*, *GBF2* and *ZCTs*, but little affected the expressions of *ORCA2* and *CrWRKY1*. Although overexpression of *CrBPF1* could not obviously increase the accumulation of TIAs and even caused the decrease of serpentine, it could extensively regulate TIA pathway genes and increase the expression of TIA transcriptional repressors, which may be a good direction to the research of TIA biosynthesis [44].

It has been reported that using G-box element in the *STR* promoters as bait to screen *C. roseus* yeast expression library resulted in identification of G-box binding factors CrGBF1 and CrGBF2 [37,45]. Both CrGBF1 and CrGBF2 act as transcriptional repressors of the *STR* via binding to the NR element in *STR* promoter, which indicated that GBFs may play an important role in the regulation of the expression of *STR* and the accumulation of TIAs.

Three Cys2/His2-type zinc finger transcription factors from *C. roseus*, ZCT1, ZCT2 and ZCT3, were isolated through a yeast one-hybrid screening system using an elicitor-responsive DB element in *TDC* promoter as bait [46]. All of them repress the activities of *STR* and *TDC* promoters in trans-activation assays, which may probably be resulted from the existence of a potent repression domain, LxLxL motif in the C-terminal region of ZCTs. In addition, the ZCT proteins can also repress the transcriptional activating activation of the ORCAs, and are found to be induced by yeast extract (YE) and JA. However, there are several differences between ZCT1, ZCT2 and ZCT3. The *STR* and *TDC* promoters binding sites of ZCT1, ZCT2 are different from one of ZCT3, and the structures of ZCT1, ZCT2 and ZCT3 are also different [46]. Furthermore, the functions of them are partially different. ZCT1 and ZCT2 act as repressors of hydroxymethylbutenyl 4-diphosphate synthase (HDS) while ZCT3 has no effect on HDS [47]. A recent study shows that silencing ZCT1 was not sufficient to increase TIA production or the expression of the TIA biosynthetic genes, which may be resulted from the remained elevated expression level of ZCT3. These results reveal that the ZCTs may play overlapping but distinct functions in TIA biosynthesis [48].

## 4. Transport of TIAs in and between Organs, Tissues, Cells and Subcellular Compartments

Tissue, cell-specific and multi-organelle-participated synthesis of specific TIAs have been extensively studied with various techniques, including in situ hybridization, immunoblot, transcriptomic analysis and GFP-fusion of metabolic enzymes or transporters. Highly complicated regulation of various regulatory and structural genes, translocation of metabolic enzymes and transport of metabolic intermediates through different tissues, cells and subcellular compartments are required for efficient biosynthesis of TIAs upon various hormonal and environmental cues [49]. Three modes of transport of alkaloids in plants have been reported: inter-organ, inter-cellular and intra-cellular [50].

The typical inter-organ alkaloids transports are for berberine and nicotine: berberine is synthesized in the root of *Coptis japonicais* and then transported to the rhizome through a long distance [51]; nicotine is produced only in the root of *Nicotiana tabacum* and transported to the leaves of the plant via the xylem [52]. In *C. roseus*, synthesis of some TIAs such as vindoline and catharanthine, mainly takes place in young leaves and stems, whereas synthesis of others such as ajmalicine and serpentine mainly occurs in roots. They also display complex inter-cellular and intra-cellular transport.

## 5. Inter-Cellular Transport Is Required for Biosynthesis of TIAs

**Figure 3:**
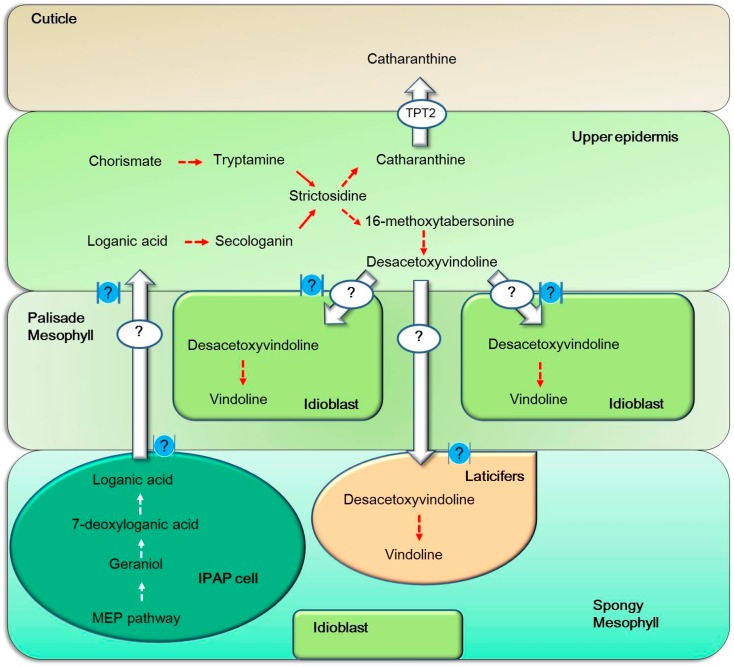
Tissue-specific biosynthesis and inter-cellular transport of TIAs through known and unknown transporters in *Catharanthus roseus.* The main TIA-biosynthesizing cells in the leaf of *C. roseus* include palisade and spongy mesophyll cells, internal phloem-associated parenchyma (IPAP), epidermal cells, laticifers and idioblasts. So far, most transporters possibly involved in transport of TIA intermediates or end products between these cells are unknown. The symbol “?” indicates the unknown transportation system of inter-cellular transport. The only identified transporter responsible for releasing of catharanthine out of upper epidermal cells onto cuticle is an ABC transporter TPT2. Whether plasmodesmata, symbolized in blue balls, are also involved in TIA intermediate transport between different types of cells remain to be determined.

Studies have revealed that TIA biosynthetic pathway in aerial organs of *C. roseus* occurs in four cell types: internal phloem-associated parenchyma (IPAP), epidermal cells, laticifers and idioblasts [53,54]. The IPAP cells exist in the periphery of stem pith or in traxylary on the upper part of the vascular bundles in leaves and are full of chloroplast with the plastid-located MEP pathway enzymes [53,55]. MEP pathway, which is required for the development and function of chloroplast, happens mostly in young green tissues [56]. The early step of seco-iridoid pathway, geraniol conversion into 10-oxogeranial via geraniol 10-hydroxylase (G10H) are co-localized in the IPAP cells of young tissues such as leaves and roots [11,57]. Iridoid oxidase (IO), 7-deoxyloganetic acid glucosyltransferase (7-DLGT) and 7-deoxyloganic acid hydroxylase (DL7H) are also localized to the IPAP cells and their transcripts, which are four times more abundant in the whole leaf than in the epidermis, while loganic acid methyl-transferase (LAMT) and secologanin synthase (SLS) are preferentially expressed in the leaf epidermis. Loganic acid, as the intermediate, is assumed to move from the IPAP cells to the epidermis to convert into secologain under the catalyzing of LAMT and SLS [58,59,60,61,62]. Tryptamine and secologanin are condensed to form strictosidine in epidermal cell of young developing shoots and leaves, meanwhile, STR- and SGD- catalyzed central steps also happen in the epidermis (Figure 3) [63]. Strictosidine is the central precursor of TIAs, some of which are directly formed in epidermis, such as ajmaline and catharanthine. Ajmaline is mainly accumulated in root epidermis, while catharanthine is mainly secreted to the surface of leaves by the ATP Binding cassette (ABC) transporter CrTPT2 to transport it from the epidermis to the leaf surface in the wax exudates (Figure 3) [64]. As for vindoline, the late precursor desacetoxyvindoline for vindoline biosynthesis is formed in epidermis, the enzymes tabersonine 16-hydroxylase 2 (T16H2), 16-hydroxytabersonine-16-*O*-methyltransferase (16OMT), T3O/T3R and *N*-methyltransferase (NMT) are preferentially localized in epidermis [31,32]. However, Desacetoxyvindoline 4-hydroxylase (D4H) and acetyl-CoA:4-*O*-deacetylvindoline 4-*O*-acetyltransferase (DAT), catalyzing the last two steps are confirmed to localize idioblasts and laticifers of leaves, stems and flowers [65]. Therefore, the intermediates desacetoxyvindoline or other accessory products need to be transported from epidermis to idioblasts or laticifers for a two-step collaboration to form vindoline [66], but whether it is necessary or how vindoline is transported out of idioblasts and laticifers remains unknown. Whether some stages of the translocation are accomplished passively through the symplasm or are controlled by plasmodesmata-localized proteins is still an open question.

## 6. Intra-Cellular Transport of TIA Intermediates and End Products

**Figure 4:**
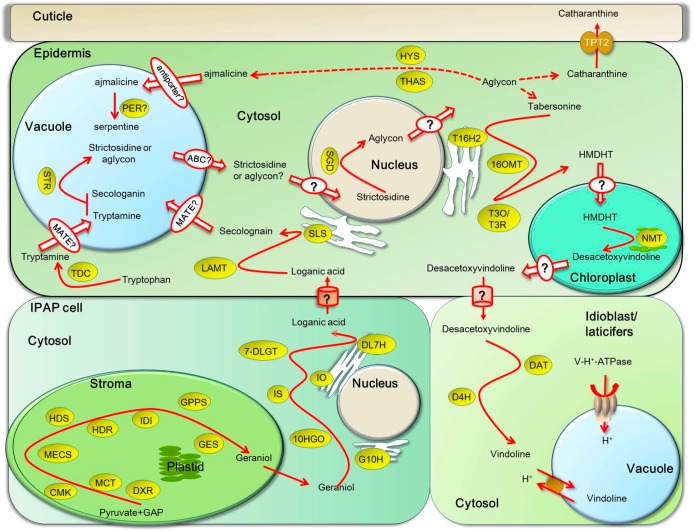
Subcellular compartmentation of TIA biosynthesis and inter- or intra-cellular transport of TIAs in *Catharanthus roseus*. TIA biosynthetic pathway in aerial organs of *C. roseus* occurs in four cell types: internal phloem-associated parenchyma (IPAP), epidermal cells, laticifers and idioblasts. An ATP-binding cassette transporters TPT2 can transport catharanthine from epidermal cells to deposit on cuticle. The transporters transport TIA intermediates between these cells, such as desacetoxyvindoline from epidermal cells to laticifers and idioblasts for vindoline biosynthesis and loganic acid communicates between the epidermal cells and IPAP cells, remain unknown, as indicated by question marks. In each type of cells, primarily, the epidermal cells, the vacuole, the chloroplast and possibly, the nucleus, are involved in the TIA biosynthesis. However, transporters or transport mechanisms for cross-membrane communication of these intermediates are largely unknown. How do two important precursors secolognain and tryptamine are transported into the vacuole for their condensation into strictosidine by vacuole-localized STR? Are they transported by MATE transporters? What is it necessary for SGD localization in the nucleus, as reported, since aglycon is still need to be transported out of the nucleus? How the product of T3O/T3R is transported into the chloroplast for methylation by the granule-localized NMT? All these questions need to be answered to give a clear scenario of intermediate transport or trafficking during the TIA biosynthesis. Abbreviations are: SGD, strictosidine β-d-glucosidase; STR, strictosidine synthase; TDC, tryptophan decarboxylase; DXS, 1-deoxy-d-xylulose 5-phosphate synthase; DXR, 1-deoxy-d-xylulose 5-phosphate reductoisomerase; MCT, MEP cytidyltransferase; CMK, 4-(cytidine 5’-diphospho)-2-*C*-methyl-d-erythritol kinase; MECS, 2-*C*-methylerythritol 2,4-cyclodiphosphate synthase; HDS, hydroxymethylbutenyl 4-diphosphate synthase; HDR, hydroxymethylbutenyl 4-diphosphate reductase; IDI, IPP isomerase; GPPS, geranyldiphosphate synthase; GES, geraniol synthase; G10H, geraniol 10-hydroxylase; 10HGO, 10-hydroxygeraniol dehydrogenase; IDS, iridoid synthase; 7DLS, 7-deoxyloganetic acid synthase; DLGT, 7-deoxyloganetic acid glucosyltransferase; DL7H, 7-deoxyloganic acid 7-hydroxylase; LAMT, loganic acid methyltransferase; SLS, secologanin synthase; T16H2, tabersonine 16-hydroxylase 2; 16OMT, 16-hydroxytabersonine-16-*O*-methyltransferase; T3O, tabersonine 3-oxygenase; T3R, tabersonine 3-reductase; NMT, *N*-methyltransferase; D4H, desacetoxyvindoline 4-hydroxylase; DAT, acetyl CoA: deacetylvindoline 4-*O*-acetyltransferase.

The MEP pathway primarily takes place in the stroma of plastids or stromules in IPAP cells [11,67]. However, isopentenyl diphosphate isomerase (IDI), which catalyzes the interconversion of isopentenyl diphosphate (IPP) and dimethylally diphosphate (DMAPP) to produce plenty of isoprenoids, was targeted to plastids, mitochondria and peroxisome [68]. Therefore, geraniol needs to be exported from the stroma by uncharacterized plastid inner or outer envelope transporter or some efficient metabolic flux into the cytosol of IPAP cells [69]. Geraniol is the substrate of the vacuolar membrane- or endoplasmic reticulum (ER)-associated G10H in the production of 10-hydroxygeraniol [70], which is further converted into the loganic acid by an ER-associated P450 DL7H. 10-hydroxygeraniol dehydrogenase (10HGO) and 7-DLGT are found in the cytosol in IPAP cells. Loganic acid and secologanin are synthesized in the cytosol of epidermal cells, since LAMT and ER-anchored P450 SLS have been ensured to locate in the cytosol of the epidermis [71]. TDC involved in the shikimate pathway is localized to the cytosol of the epidermis. Tryptamine and secologanin are transported by unidentified transporters from cytosol into the vacuole, in which STR condenses them into strictosidine [63]. The vacuole-accumulated strictosidine or its hydrolyzed product aglycon needs to be transported out of the vacuole into the cytosol. SGD was a highly stable supramolecular complex within the nucleus, indicating that transportation of strictosidine across the tonoplast by unidentified transporter plays a critical role in controlling TIA biosynthesis [63]. Strictosidine aglycone is converted into TIAs, such as catharanthine and tabersonine in the cytosol of epidermis. The conversion of tabersonine to vindoline occurs in various compartments. The first two steps catalyzed by T16H and 16OMT were localized in the cytosol [72]. T16H is an ER-anchored P450 and can release 16-hydroxytabersonine to the cytosol via its exposing catalytic site toward the cytosol [73,74]. NME is likely to be localized in the leaf epidermis in association with chloroplast thylakoid membranes [75,76,77] (Figure 4).

Laticifers and idioblasts are the cells located in stems and leaves where tabersonine derivative converts into vindoline. The last two vindoline synthetic enzymes DAT and D4H are localized in cytoplasm and nucleus of the laticifers and idioblasts cells [74]. Vindoline is formed in the cytosol of laticifers and idioblasts and transported into the vacuole by a specific proton antiporter, which is energized by the V-H^+^-ATPase. Catharanthine and AVLB are also taken up into the vacuole of epidermal cells by an uncharacterized antiporter through an H^+^-dependent mechanism (Figure 4) [78]. Since catharanthine and vindoline are not in the vacuole of the same cells in *C. roseus* leaves, they are coupled to form bisindole alkaloids vinblastine and vincristine, only because stimulation from external environment could break down the spatial separation. However, as long as bisindole alkaloid forms, they are transported and accumulated in the vacuoles.

## 7. The Biochemical, Molecular Biological Aspects of TIA Transporters

TIAs present in *C. roseus* tissues for many physiological functions. Catharanthine can inhibit the growth of fungal zoospores and shows insect toxicity at physiological concentrations on the surface of *C. roseus* leaves. The complex developmental, environmental, hormonal, organ- and cell-specific cues regulate the genes involved in the biosynthesis of TIAs [77]. Protein kinase cascade- or calcium-mediated signaling is important for the regulation of methyl jasmonate-dependent and yeast elicitor-induced TIA biosynthesis in *C. roseus* cells [79,80,81]. These factors-induced de novo biosynthesis and active secretion of TIAs cost lots of energy. The long-distance transport of end products or secretion of TIAs to the target sites also requires energy [64]. The vacuolar sequestration of these TIAs more likely depends on secondary transporters, such as MATE or multidrug resistance transporter [78].

The biosynthesis of intermediates as well as end-products of TIAs happens in different tissues and organs, and they are subsequently transported to the sites for the next reactions, storage, or for their physiological functions. Therefore, the efficient long-distance transport is essentially required. Usually, three basic transport or trafficking processes exist for organic compounds in plant cells, membrane vesicle trafficking of substances, protein-aided transport/subcellular trafficking and membrane transporters-mediated across-membrane transport [3,82].

Although great attention was paid to the transport of TIAs long time ago, now it is recognized that a passive diffusion is usually impossible for transport of highly charged TIAs, likewise for an unspecific ion-trap mechanism [20,83]. Plenty of evidence indicates that many alkaloids such as berberine in *Coptis japonica*, nicotine in tobacco, or terpenoids are transported across the plasma membrane by ABC transporters. For example, CjMDR1 expressed in the xylem of the rhizome [51] and CjABCB2 expressed in cells around the xylem of the rhizome [84] are involved in the transport of berberine to the place where berberine is synthesized; NtNUP—a plasma membrane nicotine—acts as a proton symporter of nicotine [85]; multidrug and toxic compound extrusion (MATE) transporters act as vacuolar sequestration of nicotine [86,87]; NpPDR1—a plasma membrane pleiotropic drug resistance-type ABC transporter in *Nicotiana plumbaginifolia*—transports diterpene sclareol antifungal compound [88]. Overexpression of *CjMDR1* in *C. roseus* cell cultures promoted a significant uptake and accumulation of ajmalicine and tetrahydroalstonine compared with control lines after feeding these alkaloids [89]. These results suggest that TIAs can be transported by such type of ABC transporter. Proteomic study on two independent cell lines with different TIAs metabolism reveal that some differentially expressed transporters possibly involved in the transport of TIAs, including seven ABCG proteins, three multidrug resistance pumps, two multidrug resistance-associated proteins, three soluble ABC transporters (ABCE and ABCF subfamilies) and one for each MATE and peptide transporter [90].

In *C. roseus*, TIAs are proved to be accumulated in the vacuoles by various techniques [78,91,92]. One study indicates that a specific proton antiport system could trap TIAs in the vacuoles of *C. roseus* mesophyll cells. The uptake of vindoline into the isolated tonoplast vesicles was dependent on ATP and H^+^ gradient across vacuolar membrane, since it was sharply suppressed by H^+^ gradient dissipators but unaffected by ABC transporter inhibitor [78]. Interestingly, catharanthine and anhydrovinblastine are also incorporated into the vacuole through an H^+^-dependent mechanism. These indicate that secondary multidrug antiporters, such as MATE, may also be involved in the vacuolar sequestration of TIAs [12].

However, little is known about the molecular identification of transporters involved in the transport of TIAs in *Catharanthus roseus*. Secretion of catharanthine is accomplished by a unique catharanthine efflux transporter (CrTPT2) [64], which is mainly expressed in the epidermis of young leaves and deposit onto leaf surfaces with wax cubicula [77]. Interestingly, CrTPT2 is reported to be closely homologous to key transporters involved in cuticle assembly by phylogenetic analysis, such as Arabidopsis AtPDR4, rice OsPDR6 and barley HvPDR6 ABC transporters [93,94]. When the plasma membrane-localized CrTPT2 was expressed in yeast cells, the yeast cell accumulated much less catharanthine than the control cells, while decreased accumulation of vindoline, tabersonine and strictosidine was not observed, which indicated that CrTPT2 is a plasma membrane catharanthine-specific efflux transporter. More interestingly, the VIGS of *CrTPT2* in plants reduced the catharanthine levels in leaf surface of emerging leaves compared to the control, while the unchanged vindoline level was observed in the *CrTPT2*-silenced *C. roseus* seedlings and the levels of 3’,4’-anhydrovinblastine increased by 30% in *CrTPT2*-silenced compared with control seedlings [64]. These data suggest that repression of catharanthine secretion out of epidermal cells could increase catharanthine levels in leaf epidermal cells and trigger an increase of dimers coupled from catharanthine-vindoline in leaves. The sequences of transporter proteins are usually conserved for their specific activities in transport of certain groups of organic compounds.

## 8. Transcriptomic Data Mining for Exploration of Putative Transcription Factors Involved in TIA Biosynthesis

**Figure 5:**
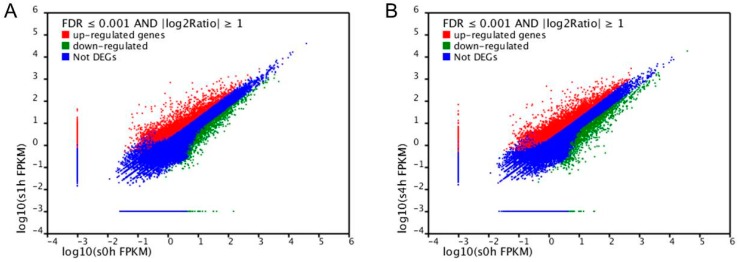
Response of the genes in *C. roseus* to MeJA treatment. (**A**) Expression of the genes at one hour after MeJA treatment; (**B**) expression of the genes at four hours after MeJA treatment.

MeJA is identified as an important signaling molecule in TIA biosynthetic pathway in many plants [95]. In order to excavate and identify transcription factors that are involved in TIA biosynthesis, we collected leaves from one month old solid plant at different time points after MeJA treatment for transcriptome analysis. According to the results of RNA-seq (Figure 5), large number of genes in *C. roseus* show differential expression after MeJA treatment, with more up-regulated genes than down-regulated expression genes. It can be inferred that there are more genes playing positive roles in MeJA-induced signal pathway than that playing negative roles.

**Figure 6:**
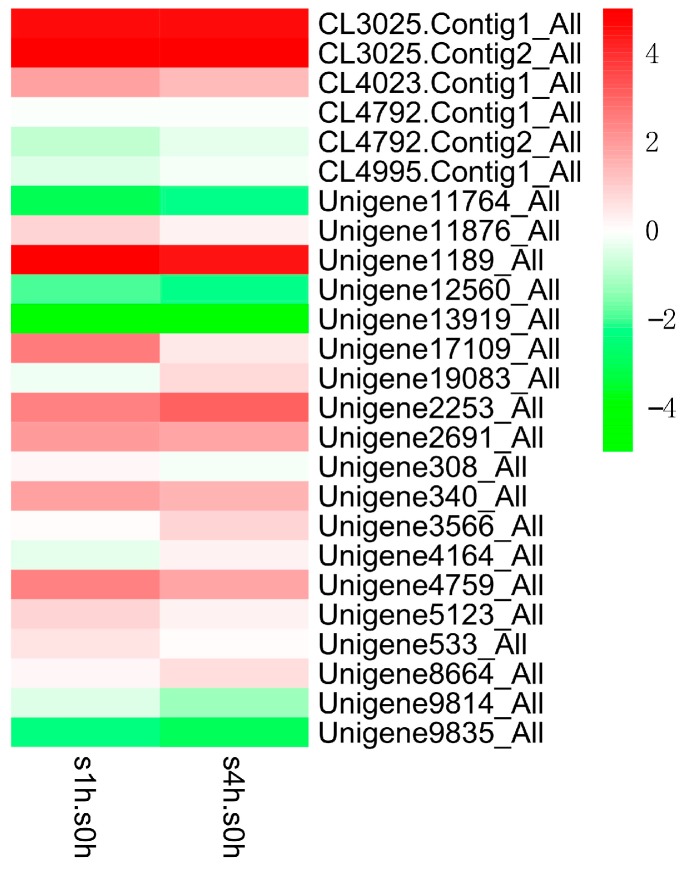
Expression patterns (log_2_ of FPKM) of AP2/ERF family in *C. roseus* after one-hour (s1h.s0h) and four-hour (s4h.s0h) treatments with MeJA.

**Figure 7:**
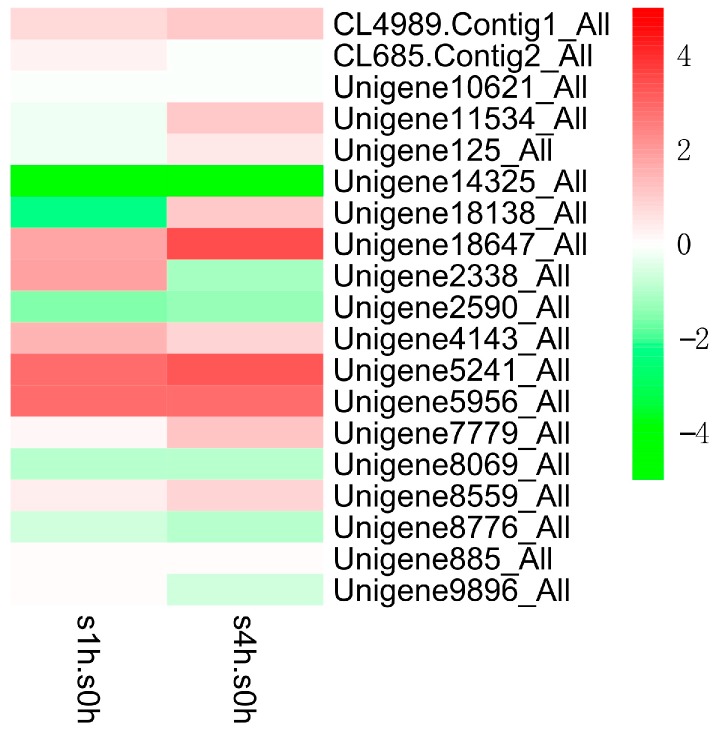
Expression patterns (log_2_ of FPKM) of bHLH family in *C. roseus* after one-hour (s1h.s0h) and four-hour (s4h.s0h) treatments with MeJA.

**Figure 8:**
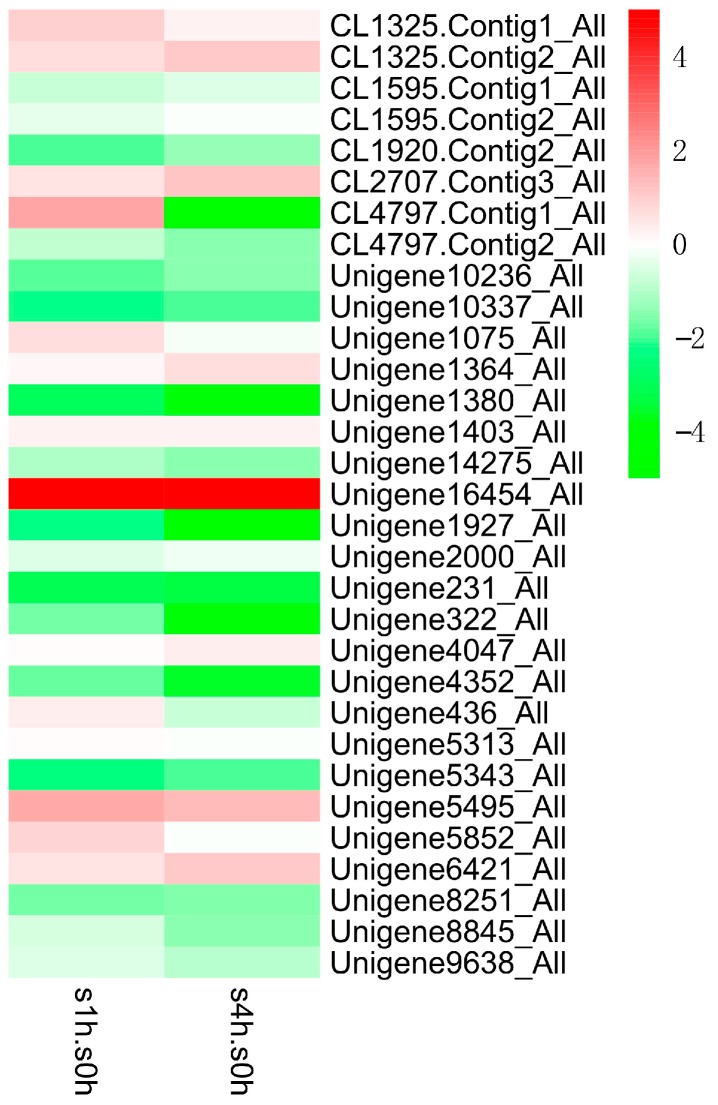
Expression patterns (log_2_ of FPKM) of WRKY family in *C. roseus* after one-hour (s1h.s0h) and four-hour (s4h.s0h) treatments with MeJA.

**Figure 9:**
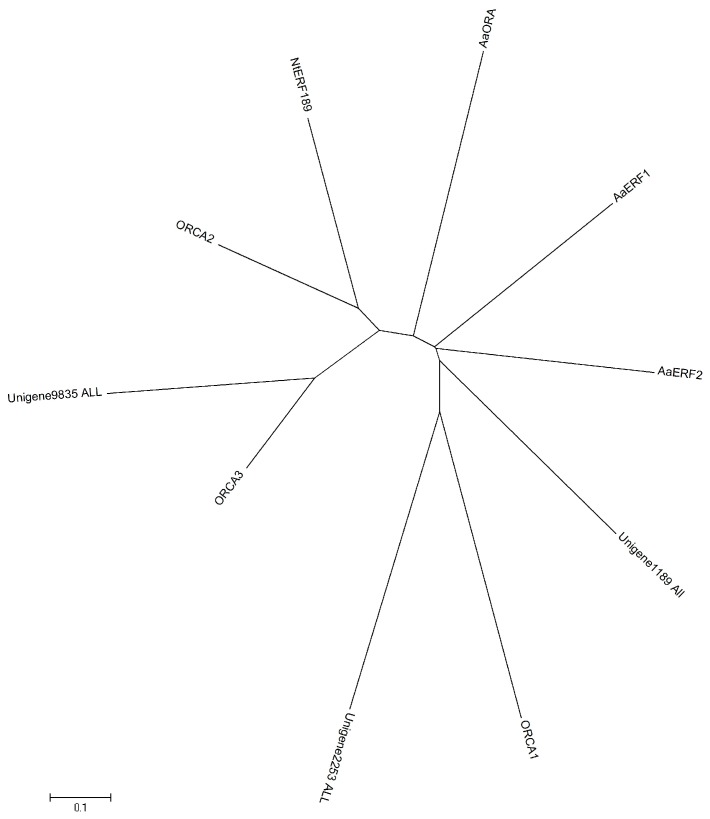
Phylogenetic analysis of three potential APETALA2/Ethylene Response Factors (AP2/ERF) transcription factors with known AP2/ERF transcription factors involved in secondary metabolite biosynthetic pathway.

In order to excavate potential transcription factors, which regulate the genes in TIA biosynthetic pathway, we further analyzed the expression patterns of different transcription factor families in *C. roseus* after MeJA treatment (Figure 6, Figure 7 and Figure 8). The results indicate that the expressions of several AP2/ERF transcription factors are significantly changed after MeJA treatment: the transcript of Unigene1189_All showed obviously increased at one hour and four hours after treatment, while the latter is slightly lower than the former, which suggested that this gene is intensively induced by MeJA in a short time and its expression can be returned to normal level slowly; the expression of Unigene2253_All gradually increased along with MeJA treatment; conversely, the expression of Unigene9835_All gradually reduced along with MeJA treatment. The three genes showing different response patterns after MeJA treatment, may encode potential transcription factors for the regulation of TIAs accumulation in *C. roseus*. Subsequently, a phylogenetic tree of the three candidate transcription factors was built with known AP2/ERF transcription factors, which are found to be involved in secondary metabolite biosynthetic pathway. Phylogenetic analysis shows the three candidates are homologous with the known AP2/ERF transcription factors involved in secondary metabolite biosynthetic pathway (Figure 9). This result further confirms the functional involvement to TIA biosynthesis of the three candidate transcription factors. Figure 7 shows the response patterns of bHLH transcription factors in *C. roseus* to MeJA. Two potential candidates that may be involved in TIA biosynthesis are revealed through this result. Unigene5241_All showed gradually increased expression while Unigene14325_All showed sharply decreased expression after MeJA treatment. The functional identification of these two bHLH transcription factors is now being carried out. As for WRKY transcription factors, most members were down-regulated by MeJA, only Unigene16454_All showed obviously increased expression after MeJA treatment (Figure 8). The role of Unigene16454_All in TIA biosynthesis as well as the functional difference between Unigene16454_All and other members in WRKY need to be studied in our future work.

## 9. Perspectives and Future Directions

As an ideal model medicinal plant, *C. roseus* actively synthesizes at least three major types of plant secondary metabolites in large amounts. Extensive studies for understanding biosynthetic pathways, regulations and subcellular and inter-cellular compartmentation of these metabolic processes so far have paved solid road toward in-depth understanding of TIA biosynthesis, transport, storage and regulation mechanisms, as well as for obtaining both basic science and application breakthroughs. Future studies may be focused on the discovery of unidentified transporters with experimental and bioinformatics methods. Those transporters may include: transporters that control the export of post tabersonine TIA intermediates from the leaf epidermis to specialized internal leaf cells where vindoline is assembled; transporters involved in the trafficking of secoiridoid precursors from specialized IPAP cells to the place where they are synthesized to the LE; transporters involved in transport of secologanin for assembly of MIAs; transporters that are involved in transport of secologanin and tryptamine into the vacuole to achieve strictosidine biosynthesis. Many outstanding questions about intra-cellular and inter-cellular transport of TIA intermediates and end products are open. Using various approaches to functionally characterize these transporters will substantially illustrate the dynamic synthesis and trafficking processes in *C. roseus* cells, which may help to draw a whole picture of subcellular and inter-cellular TIA biosynthesis.

In addition, the availability of several *C. roseus* transcriptomic databases, such as Medicinal Plant Genomics Resource (http://medicinalplantgenomics.msu.edu/) [12] and PhytoMetaSyn (http://www.phytometasyn.ca/index.php/) [96,97] could also significantly promote the research on either molecular biology or biochemical pathways in *C. roseus*. Based on these available transcriptomic databases, a genome assembly for *C. roseus* was generated to provide a near-comprehensive representation of the genic space, revealing the genomic context of key points in the TIA biosynthetic pathways, including physically clustered genes, tandem gene duplication, expression sub-functionalization and putative neo-functionalization. In addition, extensive proteomic analysis and transcriptome sequencing of *C. roseus* have been conducted recently by several groups, which provides numerous insights into the understanding of biosynthesis of complex TIAs [90,98]. These comprehensive datasets and data mining may facilitate revealing more potential regulatory networks and critical transporters for our understanding the dynamic TIA production processes in *C. roseus*.

## References

[1] Zhao J., Dixon R.A. (2009). Mate transporters facilitate vacuolar uptake of epicatechin 3′-*O*-glucoside for proanthocyanidin biosynthesis in *Medicago truncatula* and *arabidopsis*. Plant Cell.

[2] Yazaki K. (2006). Abc transporters involved in the transport of plant secondary metabolites. FEBS Lett..

[3] Zhao J. (2015). Flavonoid transport mechanisms: How to go, and with whom. Trends Plant Sci..

[4] Van Der Heijden R., Jacobs D., Snoeijer W., Hallard D., Verpoorte R. (2004). The catharanthus alkaloids: Pharmacognosy and biotechnology. Curr. Med. Chem..

[5] Leveque D., Jehl F. (2007). Molecular pharmacokinetics of catharanthus (vinca) alkaloids. J. Clin. Pharmacol..

[6] Chen Q., Zhang W., Zhang Y., Chen J., Chen Z. (2013). Identification and quantification of active alkaloids in *Catharanthus roseus* by liquid chromatography-ion trap mass spectrometry. Food Chem..

[7] Liu D.H., Jin H.B., Chen Y.H., Cui L.J., Ren W.W., Gong Y.F., Tang K.X. (2007). Terpenoid indole alkaloids biosynthesis and metabolic engineering in *Catharanthus roseus*. J. Integr. Plant Biol..

[8] Memelink J., Gantet P. (2007). Transcription factors involved in terpenoid indole alkaloid biosynthesis in *Catharanthus roseus*. Phytochem. Rev..

[9] Peebles C.A., Sander G.W., Hughes E.H., Peacock R., Shanks J.V., San K.Y. (2011). The expression of 1-deoxy-d-xylulose synthase and geraniol-10-hydroxylase or anthranilate synthase increases terpenoid indole alkaloid accumulation in *Catharanthus roseus* hairy roots. Metab. Eng..

[10] Yang C.Q., Fang X., Wu X.M., Mao Y.B., Wang L.J., Chen X.Y. (2012). Transcriptional regulation of plant secondary metabolism. J. Integr. Plant Biol..

[11] Courdavault V., Papon N., Clastre M., Giglioli-Guivarc’h N., St-Pierre B., Burlat V. (2014). A look inside an alkaloid multisite plant: The catharanthus logistics. Curr. Opin. Plant Biol..

[12] Kellner F., Kim J., Clavijo B.J., Hamilton J.P., Childs K.L., Vaillancourt B., Cepela J., Habermann M., Steuernagel B., Clissold L. (2015). Genome-guided investigation of plant natural product biosynthesis. Plant J..

[13] Hughes E.H., Hong S.B., Gibson S.I., Shanks J.V., San K.Y. (2004). Metabolic engineering of the indole pathway in *Catharanthus roseus* hairy roots and increased accumulation of tryptamine and serpentine. Metab. Eng..

[14] Chang K., Qiu F., Chen M., Zeng L., Liu X., Yang C., Lan X., Wang Q., Liao Z. (2014). Engineering the mep pathway enhanced ajmalicine biosynthesis. Biotechnol. Appl. Biochem..

[15] Krithika R., Srivastava P.L., Rani B., Kolet S.P., Chopade M., Soniya M., Thulasiram H.V. (2015). Characterization of 10-hydroxygeraniol dehydrogenase from *Catharanthus roseus* reveals cascaded enzymatic activity in iridoid biosynthesis. Sci. Rep..

[16] Geu-Flores F., Sherden N.H., Courdavault V., Burlat V., Glenn W.S., Wu C., Nims E., Cui Y., O’Connor S.E. (2012). An alternative route to cyclic terpenes by reductive cyclization in iridoid biosynthesis. Nature.

[17] Miettinen K., Dong L., Navrot N., Schneider T., Burlat V., Pollier J., Woittiez L., van der Krol S., Lugan R., Ilc T. (2014). The seco-iridoid pathway from *Catharanthus roseus*. Nat. Commun..

[18] Geerlings A., Ibañez M.M.-L., Memelink J., van der Heijden R., Verpoorte R. (2000). Molecular cloning and analysis of strictosidine β-d-glucosidase, an enzyme in terpenoid indole alkaloid biosynthesis in *Catharanthus roseus*. J. Biol. Chem..

[19] Stavrinides A., Tatsis E.C., Caputi L., Foureau E., Stevenson C.E., Lawson D.M., Courdavault V., O’Connor S.E. (2016). Structural investigation of heteroyohimbine alkaloid synthesis reveals active site elements that control stereoselectivity. Nat. Commun..

[20] Blom T., Sierra M., van Vliet T., Franke-van Dijk M., de Koning P., van Iren F., Verpoorte R., Libbenga K. (1991). Uptake and accumulation of ajmalicine into isolated vacuoles of cultured cells of *Catharanthus roseus* (L.) G. Don. And its conversion into serpentine. Planta.

[21] El-Sayed M., Choi Y.H., Frederich M., Roytrakul S., Verpoorte R. (2004). Alkaloid accumulation in *Catharanthus roseus* cell suspension cultures fed with stemmadenine. Biotechnol. Lett..

[22] Costa M.M., Hilliou F., Duarte P., Pereira L.G., Almeida I., Leech M., Memelink J., Barcelo A.R., Sottomayor M. (2008). Molecular cloning and characterization of a vacuolar class III peroxidase involved in the metabolism of anticancer alkaloids in *Catharanthus roseus*. Plant Physiol..

[23] Zhao J., Verpoorte R. (2007). Manipulating indole alkaloid production by *Catharanthus roseus* cell cultures in bioreactors: From biochemical processing to metabolic engineering. Phytochem. Rev..

[24] Goossens A., Mertens J., Pollier J., Bossche R.V., López-Vidriero I., Franco-Zorrilla J.M. (2016). The bhlh transcription factors *TSAR1* and *TSAR2* regulate triterpene saponin biosynthesis in *Medicago truncatula*. Plant Physiol..

[25] Mishra S., Phukan U.J., Tripathi V., Singh D.K., Luqman S., Shukla R.K. (2015). PsAP2 an AP2/ERF family transcription factor from *Papaver somniferum* enhances abiotic and biotic stress tolerance in transgenic tobacco. Plant Mol. Biol..

[26] Kim Y.B., Li X., Kim S.J., Kim H.H., Lee J., Kim H., Park S.U. (2013). Myb transcription factors regulate glucosinolate biosynthesis in different organs of chinese cabbage (*Brassica rapa* ssp. *Pekinensis*). Molecules.

[27] Yu Z.X., Li J.X., Yang C.Q., Hu W.L., Wang L.J., Chen X.Y. (2012). The jasmonate-responsive Ap2/ERF transcription factors AaERF1 and AaERF2 positively regulate artemisinin biosynthesis in *Artemisia annua* L.. Mol. Plant..

[28] Menke F.L., Champion A., Kijne J.W., Memelink J. (1999). A novel jasmonate-and elicitor-responsive element in the periwinkle secondary metabolite biosynthetic gene *STR* interacts with a jasmonate-and elicitor-inducible AP2-domain transcription factor, ORCA2. EMBO J..

[29] Li C.Y., Leopold A.L., Sander G.W., Shanks J.V., Zhao L., Gibson S.I. (2013). The ORCA2 transcription factor plays a key role in regulation of the terpenoid indole alkaloid pathway. BMC Plant Biol..

[30] Liu D.-H., Ren W.-W., Cui L.-J., Zhang L.-D., Sun X.-F., Tang K.-X. (2011). Enhanced accumulation of catharanthine and vindoline in *Catharanthus roseus* hairy roots by overexpression of transcriptional factor ORCA2. Afr. J. Biotechnol..

[31] Van der Fits L., Memelink J. (2000). ORCA3, a jasmonate-responsive transcriptional regulator of plant primary and secondary metabolism. Science.

[32] Peebles C.A., Hughes E.H., Shanks J.V., San K.Y. (2009). Transcriptional response of the terpenoid indole alkaloid pathway to the overexpression of *ORCA3* along with jasmonic acid elicitation of *Catharanthus roseus* hairy roots over time. Metab. Eng..

[33] Sun J., Peebles C.A. (2016). Engineering overexpression of *ORCA3* and strictosidine glucosidase in *Catharanthus roseus* hairy roots increases alkaloid production. Protoplasma.

[34] Pan Q., Wang Q., Yuan F., Xing S., Zhao J., Choi Y.H., Verpoorte R., Tian Y., Wang G., Tang K. (2012). Overexpression of ORCA3 and G10H in *Catharanthus roseus* plants regulated alkaloid biosynthesis and metabolism revealed by NMR-metabolomics. PLoS ONE.

[35] Wang M., Zi J., Zhu J., Chen S., Wang P., Song L., Yu R. (2016). Artemisinic acid serves as a novel ORCA3 inducer to enhance biosynthesis of terpenoid indole alkaloids in *Catharanthus roseus* cambial meristematic cells. Nat. Prod. Commun..

[36] Pandey S.S., Singh S., Babu C.S., Shanker K., Srivastava N.K., Shukla A.K., Kalra A. (2016). Fungal endophytes of *Catharanthus roseus* enhance vindoline content by modulating structural and regulatory genes related to terpenoid indole alkaloid biosynthesis. Sci. Rep..

[37] Pré M., Sibéril Y., Memelink J., Champion A., Doireau P., Gantet P. (2000). Isolation by the yeast one-hybrid system of cdnas encoding transcription factors that bind to the g-box element of the strictosidine synthase gene promoter from *Catharanthus roseus*. Int. J. Biochromatogr..

[38] Montiel G., Zarei A., Korbes A.P., Memelink J. (2011). The jasmonate-responsive element from the *ORCA3* promoter from *Catharanthus roseus* is active in arabidopsis and is controlled by the transcription factor AtMYC2. Plant Cell Physiol..

[39] Zhang H., Hedhili S., Montiel G., Zhang Y., Chatel G., Pré M., Gantet P., Memelink J. (2011). The basic helix-loop-helix transcription factor CrMYC2 controls the jasmonate-responsive expression of the *ORCA* genes that regulate alkaloid biosynthesis in *Catharanthus roseus*. Plant J..

[40] Van Moerkercke A., Steensma P., Schweizer F., Pollier J., Gariboldi I., Payne R., Bossche R.V., Miettinen K., Espoz J., Purnama P.C. (2015). The bhlh transcription factor bis1 controls the iridoid branch of the monoterpenoid indole alkaloid pathway in *Catharanthus roseus*. Proc. Natl. Acad. Sci. USA.

[41] Van Moerkercke A., Steensma P., Gariboldi I., Espoz J., Purnama P.C., Schweizer F., Miettinen K., Vanden Bossche R., De Clercq R., Memelink J. (2016). The basic helix-loop-helix transcription factor BIS2 is essential for monoterpenoid indole alkaloid production in the medicinal plant *Catharanthus roseus*. Plant J..

[42] Paul P., Singh S.K., Patra B., Sui X., Pattanaik S., Yuan L. (2016). A differentially regulated AP2/ERF transcription factor gene cluster acts downstream of a map kinase cascade to modulate terpenoid indole alkaloid biosynthesis in *Catharanthus roseus*. New Phytol..

[43] Suttipanta N., Pattanaik S., Kulshrestha M., Patra B., Singh S.K., Yuan L. (2011). The transcription factor CrWRKY1 positively regulates the terpenoid indole alkaloid biosynthesis in *Catharanthus roseus*. Plant Physiol..

[44] Li C.Y., Leopold A.L., Sander G.W., Shanks J.V., Zhao L., Gibson S.I. (2015). CrBPF1 overexpression alters transcript levels of terpenoid indole alkaloid biosynthetic and regulatory genes. Front. Plant Sci..

[45] Sibéril Y., Benhamron S., Memelink J., Giglioli-Guivarc’h N., Thiersault M., Boisson B., Doireau P., Gantet P. (2001). *Catharanthus roseus* g-box binding factors 1 and 2 act as repressors of strictosidine synthase gene expression in cell cultures. Plant Mol. Biol..

[46] Pauw B., Hilliou F.A., Martin V.S., Chatel G., de Wolf C.J., Champion A., Pré M., van Duijn B., Kijne J.W., van der Fits L. (2004). Zinc finger proteins act as transcriptional repressors of alkaloid biosynthesis genes in *Catharanthus roseus*. J. Biol. Chem..

[47] Chebbi M., Ginis O., Courdavault V., Glevarec G., Lanoue A., Clastre M., Papon N., Gaillard C., Atanassova R., St-Pierre B. (2014). ZCT1 and ZCT2 transcription factors repress the activity of a gene promoter from the methyl erythritol phosphate pathway in madagascar periwinkle cells. J. Plant Physiol..

[48] Rizvi N.F., Weaver J.D., Cram E.J., Lee-Parsons C.W. (2016). Silencing the transcriptional repressor, ZCT1, illustrates the tight regulation of terpenoid indole alkaloid biosynthesis in *catharanthus roseus* hairy roots. PLoS ONE.

[49] Shitan N., Kato K., Shoji T. (2014). Alkaloid transporters in plants. Plant Biotechnol..

[50] Shitan N., Yazaki K. (2007). Accumulation and membrane transport of plant alkaloids. Curr. Pharm. Biotechnol..

[51] Shitan N., Bazin I., Dan K., Obata K., Kigawa K., Ueda K., Sato F., Forestier C., Yazaki K. (2003). Involvement of cjmdr1, a plant multidrug-resistance-type atp-binding cassette protein, in alkaloid transport in *Coptis japonica*. Proc. Natl. Acad. Sci. USA.

[52] Shoji T., Hashimoto T. (2013). Smoking out the masters: Transcriptional regulators for nicotine biosynthesis in tobacco. Plant Biotechnol..

[53] Burlat V., Oudin A., Courtois M., Rideau M., St-Pierre B. (2004). Co-expression of three mep pathway genes and *geraniol 10-hydroxylase* in internal phloem parenchyma of *Catharanthus roseus* implicates multicellular translocation of intermediates during the biosynthesis of monoterpene indole alkaloids and isoprenoid-derived primary metabolites. Plant J..

[54] Verma P., Mathur A.K., Srivastava A., Mathur A. (2012). Emerging trends in research on spatial and temporal organization of terpenoid indole alkaloid pathway in *Catharanthus roseus*: A literature update. Protoplasma.

[55] Mahroug S., Burlat V., St-Pierre B. (2007). Cellular and sub-cellular organisation of the monoterpenoid indole alkaloid pathway in *Catharanthus roseus*. Phytochem. Rev..

[56] Gutierrez-Nava Mde L., Gillmor C.S., Jimenez L.F., Guevara-Garcia A., Leon P. (2004). *Chloroplast biogenesis* genes act cell and noncell autonomously in early chloroplast development. Plant Physiol..

[57] De Luca V., Salim V., Thamm A., Masada S.A., Yu F. (2014). Making iridoids/secoiridoids and monoterpenoid indole alkaloids: Progress on pathway elucidation. Curr. Opin. Plant Biol..

[58] Salim V., Wiens B., Masada-Atsumi S., Yu F., De Luca V. (2014). 7-deoxyloganetic acid synthase catalyzes a key 3 step oxidation to form 7-deoxyloganetic acid in *Catharanthus roseus* iridoid biosynthesis. Phytochemistry.

[59] Salim V., Yu F., Altarejos J., Luca V. (2013). Virus-induced gene silencing identifies *Catharanthus roseus* 7-deoxyloganic acid-7-hydroxylase, a step in iridoid and monoterpene indole alkaloid biosynthesis. Plant J..

[60] Irmler S., Schröder G., St-Pierre B., Crouch N.P., Hotze M., Schmidt J., Strack D., Matern U., Schröder J. (2000). Indole alkaloid biosynthesis in *Catharanthus roseus*: New enzyme activities and identification of cytochrome p450 cyp72a1 as secologanin synthase. Plant J..

[61] Murata J., Roepke J., Gordon H., de Luca V. (2008). The leaf epidermome of *Catharanthus roseus* reveals its biochemical specialization. Plant Cell.

[62] Asada K., Salim V., Masada-Atsumi S., Edmunds E., Nagatoshi M., Terasaka K., Mizukami H., De Luca V. (2013). A 7-deoxyloganetic acid glucosyltransferase contributes a key step in secologanin biosynthesis in madagascar periwinkle. Plant Cell.

[63] Guirimand G., Courdavault V., Lanoue A., Mahroug S., Guihur A., Blanc N., Giglioli-Guivarc’h N., St-Pierre B., Burlat V. (2010). Strictosidine activation in apocynaceae: Towards a “nuclear time bomb”?. BMC Plant Biol..

[64] Yu F., De Luca V. (2013). Atp-binding cassette transporter controls leaf surface secretion of anticancer drug components in *Catharanthus roseus*. Proc. Natl. Acad. Sci. USA.

[65] St-Pierre B., Vazquez-Flota F.A., De Luca V. (1999). Multicellular compartmentation of *Catharanthus roseus* alkaloid biosynthesis predicts intercellular translocation of a pathway intermediate. Plant Cell.

[66] Qu Y., Easson M.L., Froese J., Simionescu R., Hudlicky T., De Luca V. (2015). Completion of the seven-step pathway from tabersonine to the anticancer drug precursor vindoline and its assembly in yeast. Proc. Natl. Acad. Sci. USA.

[67] Oudin A., Mahroug S., Courdavault V., Hervouet N., Zelwer C., Rodríguez-Concepción M., St-Pierre B., Burlat V. (2007). Spatial distribution and hormonal regulation of gene products from methyl erythritol phosphate and monoterpene-secoiridoid pathways in *Catharanthus roseus*. Plant Mol. Biol..

[68] Guirimand G., Guihur A., Phillips M.A., Oudin A., Glevarec G., Melin C., Papon N., Clastre M., St-Pierre B., Rodriguez-Concepcion M. (2012). A single gene encodes isopentenyl diphosphate isomerase isoforms targeted to plastids, mitochondria and peroxisomes in *Catharanthus roseus*. Plant Mol. Biol..

[69] Simkin A.J., Miettinen K., Claudel P., Burlat V., Guirimand G., Courdavault V., Papon N., Meyer S., Godet S., St-Pierre B. (2013). Characterization of the plastidial geraniol synthase from madagascar periwinkle which initiates the monoterpenoid branch of the alkaloid pathway in internal phloem associated parenchyma. Phytochemistry.

[70] Guirimand G., Burlat V., Oudin A., Lanoue A., St-Pierre B., Courdavault V. (2009). Optimization of the transient transformation of *Catharanthus roseus* cells by particle bombardment and its application to the subcellular localization of hydroxymethylbutenyl 4-diphosphate synthase and geraniol 10-hydroxylase. Plant Cell Rep..

[71] Guirimand G., Guihur A., Ginis O., Poutrain P., Hericourt F., Oudin A., Lanoue A., St-Pierre B., Burlat V., Courdavault V. (2011). The subcellular organization of strictosidine biosynthesis in *Catharanthus roseus* epidermis highlights several trans-tonoplast translocations of intermediate metabolites. FEBS J..

[72] Murata J., Luca V.D. (2005). Localization of tabersonine 16-hydroxylase and 16-oh tabersonine-16-*O*-methyltransferase to leaf epidermal cells defines them as a major site of precursor biosynthesis in the vindoline pathway in *Catharanthus roseus*. Plant J..

[73] Besseau S., Kellner F., Lanoue A., Thamm A.M., Salim V., Schneider B., Geu-Flores F., Höfer R., Guirimand G., Guihur A. (2013). A pair of tabersonine 16-hydroxylases initiates the synthesis of vindoline in an organ-dependent manner in *Catharanthus roseus*. Plant Physiol..

[74] Guirimand G., Guihur A., Poutrain P., Héricourt F., Mahroug S., St-Pierre B., Burlat V., Courdavault V. (2011). Spatial organization of the vindoline biosynthetic pathway in *Catharanthus roseus*. J. Plant Physiol..

[75] Dethier M., De Luca V. (1993). Partial purification of an n-methyltransferase involved in vindoline biosynthesis in *Catharanthus roseus*. Phytochemistry.

[76] Roytrakul S., Verpoorte R. (2007). Role of vacuolar transporter proteins in plant secondary metabolism: *Catharanthus roseus* cell culture. Phytochem. Rev..

[77] Roepke J., Salim V., Wu M., Thamm A.M., Murata J., Ploss K., Boland W., de Luca V. (2010). Vinca drug components accumulate exclusively in leaf exudates of madagascar periwinkle. Proc. Natl. Acad. Sci. USA.

[78] Carqueijeiro I., Noronha H., Duarte P., Gerós H., Sottomayor M. (2013). Vacuolar transport of the medicinal alkaloids from *Catharanthus roseus* is mediated by a proton-driven antiport. Plant Physiol..

[79] Zhao J., Hu Q., Guo Y.Q., Zhu W.H. (2001). Elicitor-induced indole alkaloid biosynthesis in *Catharanthus roseus* cell cultures is related to Ca^2+^ influx and the oxidative burst. Plant Sci..

[80] Lee-Parsons C.W., Ertürk S. (2005). Ajmalicine production in methyl jasmonate-induced *catharanthus roseus* cell cultures depends on Ca^2+^ level. Plant Cell Rep..

[81] Zhao J., Davis L.C., Verpoorte R. (2005). Elicitor signal transduction leading to production of plant secondary metabolites. Biotechnol. Adv..

[82] Zhao J., Dixon R.A. (2010). The ‘ins’ and ‘outs’ of flavonoid transport. Trends Plant Sci..

[83] Wink M., Witte L. (1993). Quinolizidine alkaloids in *Genista*
*acanthoclada* and its holoparasite, *Cuscuta palaestina*. J. Chem. Ecol..

[84] Shitan N., Dalmas F., Dan K., Kato N., Ueda K., Sato F., Forestier C., Yazaki K. (2013). Characterization of *Coptis japonica* CjABCB2, an at p-binding cassette protein involved in alkaloid transport. Phytochemistry.

[85] Hildreth S.B., Gehman E.A., Yang H., Lu R.H., Ritesh K., Harich K.C., Yu S., Lin J., Sandoe J.L., Okumoto S. (2011). Tobacco nicotine uptake permease (NUP1) affects alkaloid metabolism. Proc. Natl. Acad. Sci. USA.

[86] Morita M., Shitan N., Sawada K., van Montagu M.C., Inzé D., Rischer H., Goossens A., Oksman-Caldentey K.M., Moriyama Y., Yazaki K. (2009). Vacuolar transport of nicotine is mediated by a multidrug and toxic compound extrusion (mate) transporter in *Nicotiana tabacum*. Proc. Natl. Acad. Sci. USA.

[87] Shoji T., Inai K., Yazaki Y., Sato Y., Takase H., Shitan N., Yazaki K., Goto Y., Toyooka K., Matsuoka K. (2009). Multidrug and toxic compound extrusion-type transporters implicated in vacuolar sequestration of nicotine in tobacco roots. Plant Physiol..

[88] Stukkens Y., Bultreys A., Grec S., Trombik T., Vanham D., Boutry M. (2005). Nppdr1, a pleiotropic drug resistance-type atp-binding cassette transporter from *Nicotiana plumbaginifolia*, plays a major role in plant pathogen defense. Plant Physiol..

[89] Pomahačová B., Dušek J., Dušková J., Yazaki K., Roytrakul S., Verpoorte R. (2009). Improved accumulation of ajmalicine and tetrahydroalstonine in *Catharanthus* cells expressing an abc transporter. J. Plant Physiol..

[90] Champagne A., Rischer H., Oksman-Caldentey K.M., Boutry M. (2012). In-depth proteome mining of cultured *Catharanthus roseus* cells identifies candidate proteins involved in the synthesis and transport of secondary metabolites. Proteomics.

[91] Deus-Neumann B., Zenk M. (1984). A highly selective alkaloid uptake system in vacuoles of higher plants. Planta.

[92] Neumann D., Krauss G., Hieke M., Gröger D. (1983). Indole alkaloid formation and storage in cell suspension cultures of *Catharanthus roseus*. Planta Med..

[93] Bessire M., Borel S., Fabre G., Carraça L., Efremova N., Yephremov A., Cao Y., Jetter R., Jacquat A.C., Métraux J.P. (2011). A member of the pleiotropic drug resistance family of atp binding cassette transporters is required for the formation of a functional cuticle in *Arabidopsis*. Plant Cell.

[94] Chen G., Komatsuda T., Ma J.F., Nawrath C., Pourkheirandish M., Tagiri A., Hu Y.G., Sameri M., Li X., Zhao X. (2011). An atp-binding cassette subfamily g full transporter is essential for the retention of leaf water in both wild barley and rice. Proc. Natl. Acad. Sci. USA.

[95] De Geyter N., Gholami A., Goormachtig S., Goossens A. (2012). Transcriptional machineries in jasmonate-elicited plant secondary metabolism. Trends Plant Sci..

[96] Facchini P.J., Bohlmann J., Covello P.S., de Luca V., Mahadevan R., Page J.E., Ro D.K., Sensen C.W., Storms R., Martin V.J. (2012). Synthetic biosystems for the production of high-value plant metabolites. Trends Biotechnol..

[97] Xiao M., Zhang Y., Chen X., Lee E.J., Barber C.J., Chakrabarty R., Desgagné-Penix I., Haslam T.M., Kim Y.B., Liu E. (2013). Transcriptome analysis based on next-generation sequencing of non-model plants producing specialized metabolites of biotechnological interest. J. Biotechnol..

[98] Verma M., Ghangal R., Sharma R., Sinha A.K., Jain M. (2014). Transcriptome analysis of *Catharanthus roseus* for gene discovery and expression profiling. PLoS ONE.

